# A Study of Stakeholder Views to Shape a Communication Strategy for GMO in Brazil

**DOI:** 10.3389/fbioe.2015.00179

**Published:** 2015-11-09

**Authors:** Deise Maria Fontana Capalbo, Olivia Márcia Nagy Arantes, Alexandre Gori Maia, Izaias Carvalho Borges, José Maria Ferreira Jardim da Silveira

**Affiliations:** ^1^Embrapa Environment, Brazilian Corporation of Agricultural Research, Jaguariúna, Brazil; ^2^Formerly affiliated with Department of General Biology, State University of Londrina, Londrina, Brazil, (Retired); ^3^Institute of Economics, State University of Campinas, Campinas, Brazil

**Keywords:** public awareness, consumer perception, GM plants, Brazilian agriculture, communication strategy

## Abstract

This paper analyzes the view of stakeholders on genetically modified organisms (GMOs) and the implications of these views on communication strategies for agricultural biotechnology in Brazil. It identifies and describes common groups of attitudes toward GMOs using multivariate statistical analyses. The study then looks for patterns of association between the common attitude groups and the following variables: socioeconomic characteristics trust in institutions as information sources and familiarity with the Brazilian biosafety authority. The article contributes to the understanding of public awareness by highlighting how information sources, trust in institutions, and socioeconomic characteristics, such as age and occupational qualification, play important roles in defining patterns of attitudes toward GMOs. The paper also discusses the implications of this knowledge for the development of a communication strategy plan that would promote public awareness and stimulate a well-informed Brazilian public debate on biosafety.

## Introduction

The development of agricultural biotechnology is a complex process that involves the participation of public research institutions, universities, biotechnology companies, corporations in the agrochemical sector, farmers, the processing industry, retail chains, and consumers. Experience with transgenic soybean crops, which were created in the mid-1990s, has shown the importance of other stakeholders who are not directly linked to the research and agribusiness supply chains, such as consumer rights advocates, environmentalists, health professionals, various members of the media, regulators, scientists, agricultural policy makers, and corporate stakeholders (Hall and Martin, 2005). Moreover, the distribution process generated new actors who were seeking business opportunities that were created with new labeling and cargo segregation requirements (Oliveira et al., 2012).

The first generation of genetically modified organisms (GMOs) was developed to meet the needs of farmers, by developing herbicide-tolerant soybeans and insect-resistant cotton and corn, for example. Studies have shown that these crops have provided economic benefits for farmers and for the industry that produces genetically modified (GM) seeds, as well as environmental benefits for society as a whole, especially in terms of the reduction of chemical pesticide usage and increased efficiency in pest control. However, very few of these benefits are recognized by end consumers (Shelton et al., 2002; Mucci and Hough, 2003; Wu, 2006; Borges et al., 2009). This asymmetry in the perceived benefits of GMOs may increase the awareness of and aversion to the risks of the technology.

Thus, one major challenge for institutions controlling the process of developing agricultural biotechnology is to determine how to reduce the asymmetry of the public’s perception of the benefits and the safety of GM crops among the various stakeholders.

In the case of GM crops, these technologies have greatly politicized issues surrounding the regulation and even the legitimacy of the use of this scientific and technological knowledge in many countries, including France, Brazil, Mexico, and Ethiopia. The polarization of the debate amplifies the public perception of the risk associated with the diffusion of the technology. Therefore, the regulation of controversial technology is characterized by the strong influence that the public’s perception of risk may have on the decisions of regulatory policy makers. This means that risk control policies can be adopted without evidence that these technologies actually cause any harm (Zilberman, 2006).

Although agricultural biotechnology is very important in Brazil, the second largest producer of GM crops in the world, public opinion regarding GMOs in this country has been investigated in just a few studies when compared to the international literature. Some of the studies are specific, such as that of Gonzalez et al. (2009) who examined the theme of accepting bio-fortified products. Preparing for the release of the transgenic bean, Embrapa (Brazilian Agricultural Research Corporation) developed a pilot experiment investigating communication and public participation regarding GMOs within the Project of Environmental and Social Assessment of Risks of Genetically Modified Organisms (Guivant et al., 2009). This project acknowledges that the transfer of knowledge does not necessarily promote or enhance the understanding of the process or product.

Studies with a wider scope (Vogt and Polino, 2003; Massarani and Moreira, 2005; Guivant, 2006) were conducted over a period of great uncertainty regarding GMOs. Furnival and Pinheiro (2008) showed in a study with focus groups that, with few exceptions, people do not know what GMOs are, but they voiced suspicion about the “ulterior motives” of the entities that “defend” GM crops. According to the authors, the public understands that where there is smoke (controversy), there is fire (malicious intent).

The products of agricultural biotechnology involve costs and benefits for a wide range of parties involved in its production, marketing, and consumption. Controversy can be interpreted as a product of different moral choices that are related to the individuals’ attitudes. According to Gaskell (2000), the variables related to the rejection of GM crops are similar to those that explain the rejection of other technologies, such as nuclear energy, stem cell research, nanotechnology, and animal cloning.

Several studies regarding risk awareness show that perceived benefits are the main explanatory variable pertaining to awareness and rejection of technological risk: the lower the perceived benefits, the greater the risk aversion, and, consequently, the greater the rejection of that technology (Starr, 1969; Slovic, 2000).

Trust is also a concept with a complex nature that impacts the perception of risk (Slovic, 1987; Lassen et al., 2002; Frewer et al., 2003). Other components include confidence in the information source, confidence in the institutions that analyze and manage the risks, the degree of familiarity with the technology, and the nature of the risk, for example, whether the risk is voluntary or involuntary, known or unknown, and individual or collective (Slovic, 1987). Other studies conclude that trust in the institutions and agents that participate in the innovative process, such as universities, research institutions, private companies, and regulatory agencies, is a decisive factor in determining consumer attitudes (Barling et al., 1999; Costa-Font et al., 2008).

When conflicting information arises concerning an issue, the values of the individual or the “subjective knowledge” will inevitably win out over other information. One well-accepted model of the formation of consumer attitudes is the “Multi-attribute Model” (Fishbein, 1963), which indicates that attitudes toward products are based not only on knowledge of the product itself but also on the attributes or values of the consumer (Costa et al., 2000; Oda and Soares, 2000). Credibility and confidence must withstand the arguments of each group, and each factor is rooted in its own value system.

Many other factors that can influence the perception and public attitudes related to GM crops should be taken in account, including socioeconomic characteristics, such as income level, education, age, and gender (Hwang et al., 2005; Gaskell et al., 2006); cultural factors, such as world views, and political and religious beliefs (Coyle et al., 2003; Scheitle, 2005; Han and Harrison, 2007; Montpetit and Rouillard, 2008); health problems, such as allergies to certain types of foods (Gaivoronskaia and Hvinden, 2006); the level of knowledge about biology and genetic engineering, which in many cases prevents consumers from becoming aware of the technology’s benefits (Grobe et al., 1999; Gaskell et al., 2006; Gurudasani and Sheth, 2009); the way that the media addresses genetic engineering issues (Bonny, 2003; McCluskey and Swinnen, 2004; Bauer, 2005); and confidence in businesses and institutions that participate in the development process and in the risk analysis technology.(House et al., 2004; Barnett et al., 2007; Peters et al., 2007).

According to Aerni (2005), public perception is strongly influenced by information from the media, whose primary sources are experts who work in different institutions, such as private companies, governments, universities, research institutes, and public interest groups, such as farmers’ associations and non-governmental organizations (NGOs) for environmental protection or the defense of consumer rights. This finding leads Aerni and Bernauer (2006) to propose that a study of the social perception of GM crops can be conducted through opinion polls that are addressed to specialists or stakeholders who influence both public opinion and policy decisions. The results show that, compared with the public, these experts perceived less risk involved in a set of seven different applications of modern biotechnology. However, both – the public and experts – judged the risks of applications in food production as greater than the risks of medical applications.

The objective of this study was to evaluate and analyze individuals’ attitudes toward biotechnology and its applications, with a focus on agricultural biotechnology in Brazil. The results were based on responses from a sample of 1439 users from the main agricultural agency in Brazil – Embrapa, which can be considered representative of a group of stakeholders in agriculture in this country. We first identified groups of relatively homogenous attitudes toward GMOs applying multivariate statistical analysis [multiple correspondence analysis (MCA) and cluster analysis (CA)]. We then analyzed the patterns of association between these groups of attitudes, socioeconomic characteristics, trusted information sources and familiarity with the Brazilian biosafety authority.

## Materials and Methods

### Survey Design

Analyses are based on data from an online survey with open-ended and multiple-choice questions. The questionnaire, translated to English and presented in Appendix 1 in Supplementary Material, was based on the Eurobarometer (Gaskell et al., 2006), documents from the National Science Foundation (2008) and the work by Vogt and Polino (2003). The variables used in the analysis are presented in Appendix 2 in Supplementary Material and they can be grouped into three main sections: (i) socioeconomic variables, (ii) perception and awareness of GMOs and biosafety, and (iii) trusted information sources and familiarity with the Brazilian biosafety authority. To test the consistency of the answers, some questions were deliberately duplicated with opposite meanings, such as “*Using transgenic plants to produce food is not harmful to the environment*” and “*Using transgenic plants to produce food is harmful to the environment*.”

The questionnaire was first validated with a group of 30 people of 18 years or older, whose level of education ranged from the fourth grade to a college degree. Next, the survey was available online on the project site^1^ and on Embrapa’s site^2^ for 6 months. It was widely disseminated among users of Embrapa’s database through emails requesting their participation in the survey.^3^ The option of receiving a paper copy of the completed survey through the mail was also available. The final sample contained 1439 answers, mostly from stakeholders involved in agriculture.

### Data Analysis

The consumers were classified into common groups of perception and awareness of GMOs, applying multivariate statistical analyses to the variables presented in Appendix 2 in Supplementary Material. The relationships among the multiple qualitative categories of these variables were analyzed using MCA and CA.

#### Multiple Correspondence Analysis

The MCA was used to reduce the information presented in a binary matrix of cross tabulations among categorical variables, determining the number of dimensions needed to better represent the relationship structure between the nominal categories. MCA is based on the technique of using principal components to simplify the data’s structure, identifying dimensions that can explain a large share of the information presented in the contingent data (Greenacre, 2007).

In MCA, distances between the categories of analysis are represented by the Chi-squared statistics, which measures the differences between the row and columns relative frequencies (profiles). The inertial represents the degree of variation among the profiles. The higher the deviation of row and column profiles from their expected (average) values, the higher the total inertial.

Based on algebraic principles, MCA decomposes the correlation structure of this matrix of distances in dimensions represented by (i) eigenvectors, which express the projection of these distances in a geometric space and (ii) eigenvalues, which express the contribution of each dimension to explain the total inertia. After identifying the key dimensions that represent the variation in the data, MCA facilitates the understanding of the structure of associations between the categories.

The geometric dispersion of the categories in the space defined by the dimensions of the MCA shows the nature of associations between the qualitative variables of the problem. Groups of categories that are close together reveal similarities in associations, whereas groups of categories that are further apart signify repulsion between them (Hoffmann and Franke, 1986). Categories that are close to the origin of a dimension (centroid) have low levels of contributions to its total inertia, i.e., their frequencies slightly differ in relation to the structure represented by the dimension.

Analyses were carried out using the CORRESP procedure of the SAS System (SAS, 2012). The main advantage of MCA is that it makes it possible to simultaneously analyze the multiple relations between the variables of interest. For example, it allows to understand to what extent the positive perception of GMOs is related to the awareness regarding the use of transgenic plants to reduce the use of pesticides and/or to produce medicines. Nonetheless, since MCA summarizes multiple heterogeneous constructs in a small number of dimensions, some sources of variability may be lost in this process. The dimensions will represent the strongest patterns of relationship. A limited number of categories were used in the questionnaire to simplify analyses and to control the sources of variability. More specifically: three categories for perception of GMOs (*Positive*, *Neutral*, *Negative*; or *Optimistic*, *Pessimistic*, *Undecided*; or *Agree*, *Disagree*, *Don’t know*); and two categories for awareness of GMOs (*Familiar*; *Unfamiliar*) were employed.

#### Cluster Analysis

Once the dimensions of the correspondence analysis were obtained, we defined common groups of associations using CA. CA is a multivariate hierarchical classification technique that distributes the observations among mutually exclusive groups, such that the characteristics are homogeneous within groups and heterogeneous between them. The clustering method adopted in this paper is that of Ward (Ketchen and Shook, 1996), which creates hierarchical groups such that the variances within groups are minimal and the variances between them are maximal. The criterion for this technique at each stage of aggregation is to find the next class that minimizes the variability within the new group. In order to better understand the contribution of the sum of squares within groups (within variability), the sum tends to be divided by the total sum of squares (total variability) to represent a maximum proportion of the variability (semi-partial *R*^2^).

First, there is 0 degree of generalization (all observations are distinct from each other) and, by the end of the process, there is 100% generalization (all observations are similar to each other). We had to choose between the number of groups required and the maximum degree of generalization acceptable, or somewhere between the two options, while examining the costs and benefits of each choice. Analyses were carried out using the CLUSTER procedure of the SAS System (SAS, 2012).

## Results

### Sample Characteristics

The sample contained 1439 answers and was predominately represented by individuals with a high level of education and living in the most developed regions; Aerni (2005) indicated that these characteristics are usually related to groups with greater autonomy in decision making.

All regions are significantly represented in our sample, with the exception of an underrepresentation of the less developed (North with 4% and Northeast with 10%) and an overrepresentation of the more developed (Southeast with 53%, South with 18%, and Midwest with 15%). For a better comprehension of such percentages, Brazil’s total population is regionally distributed as follows: (a) North (8.3%), (b) Northeast (27.9%), (c) Southeast (42.0%), (d) South (14.3%), and (e) Midwest (7.4%), according to the Brazilian Institute of Geography and Statistics (IBGE).^4^

Respondents with a college degree made up 88% of the sample and those with primary education made up just 1%, as presented in Table 2. Women (46%) and men (54%) were almost equally distributed, with the prevalence of adults between 25 and 34 years old (34%) and highly qualified professional workers (65%).

Most respondents declared familiarity with the Brazilian biosafety authority (72%), albeit just half knew the name of this authority (see Table 3). Results also highlighted that respondents have more confidence in information provided by scientists and experts (research and educational institutions). The percentage of people who trusted scientists and experts (77% for information on GMOs and 74% for information on biosafety) was much higher than those shown in relation to NGOs (with 39% for GMOs and 37% for biosafety), the Government (29% for GMOs and 25% for biosafety), private corporations (16% for GMOs and 13% for biosafety), and the media (14% for GMOs and 12% for biosafety).

However, the sources of information that respondents “had heard talk about transgenic plants and biosafety” were (1) Media (TV, radio, and magazines, with 84%), (2) Experts (scientists and specialists, with 73%), and (3) NGOs (67%). In other words, according to the respondents, the media is the main source of information despite being a less knowledgeable source.

### Attitudes Toward GMOs

Table 1 presents the percentage distribution of responses in relation to the perception of and knowledge surrounding GMOs. Interviewees tend to view the terms “biotechnology,” “biosafety,” and “genetic engineering” more positively. The percentage of positive responses for these questions was, respectively, 81, 75, and 70%. On the other hand, just 38% showed positive attitudes toward the term “transgenic plant” and 39% showed positive attitudes toward the term “genetically modified organisms.”

**Table 1:** **Percentage of answers regarding perception and awareness of GMOs and biosafety**.

Question	Positive	Neutral	Negative
Perception of biotechnology	81.4	8.0	10.6
Perception of biosafety	75.1	12.6	12.3
Perception of transgenic plants (TP)	38.2	41.2	20.6
Perception of genetically modified organisms	39.4	39.0	21.6
Perception of genetic engineering	70.4	12.4	17.2

	**Familiar**	**Unfamiliar**	

The use of transgenic plants to produce medicine	54.8	45.2	
The use of transgenic plants to produce food	94.7	5.4	

	**Optimistic**	**Pessimistic**	**Undecided**

The use of transgenic plants to produce medicine	51.9	22.4	25.7
The use of transgenic plants to produce food	44.1	38.3	17.7

	**Agree**	**Disagree**	**Don’t know**

The use of TP to produce medicine is not harmful to the environment	28.2	40.2	31.6
The use of TP to produce medicine is not harmful to human health	31.6	29.8	38.6
The use of TP to produce medicine is harmful to the environment	40.2	30.8	29.0
The use of TP to produce medicine is harmful to human health	27.2	34.0	38.8
The use of TP to produce medicine is ethically acceptable	49.6	27.9	22.5

	**Agree**	**Disagree**	**Don’t know**

The use of TP to produce food is not harmful to the environment	26.8	50.0	23.2
The use of TP to produce food is not harmful to human health	29.7	42.8	27.5
The use of TP to produce food is harmful to the environment	48.2	31.1	20.7
The use of TP to produce food is harmful to human health	41.0	33.2	25.9
The use of TP to produce food is ethically acceptable	45.4	37.2	17.4

The majority of respondents (95%) were familiar with the use of transgenic plants to produce food, but just 55% were familiar with the use to produce medicines. However, respondents have a more optimistic view of transgenic plants being used to produce medicine (52% of the responses) than being used to produce food (44%).

Transgenic plants for use as a medicine were perceived as being of higher risk than transgenic plants used as food, especially risks related to the environment. Almost half of the respondents (48%) agreed that using transgenic plants to produce food is harmful to the environment (40% for the use to produce medicine) and 45% agreed that its use to produce food is ethically acceptable (50% agreed with use to produce medicine).

### Groups of Attitudes

We generated groups of respondents that were relatively homogenous regarding perception and awareness of GMOs and biosafety. This was done so by first applying MCA to the complete set of variables presented in Table 1. Questions with opposite meanings were deliberately used in the MCA as explained in Section “Survey Design.” It was expected that the questions would present high and negative association. Inconsistent responses were then identified and analyzed separately in the next steps (classification) of the multivariate analysis.

We selected the three principal dimensions of the MCA with the highest contributions to the total inertia. Based on the contribution of the categories of analysis to the inertial of each dimension, these dimensions are interpreted as follows:

*Dimension 1* (28% of the total inertia): the categories that most contribute to the inertial of this dimension are the following: *a positive or negative attitude toward transgenic plants and genetically modified organisms*; *optimism or pessimism regarding the use of transgenic plants to produce medicine or food*; *agreement or disagreement regarding the effects on the environment and human health*, *ethics, and the use of transgenic plants to produce medicines or food*. This result confirms that the public’s perception of transgenic crops contributes crucially to the inertia of the most important dimension.
*Dimension 2* (16% of the total inertia): the categories that most contribute to the inertial of this dimension are *neutrality with regard to transgenic plants and genetically modified organisms* and *an unawareness that the use of transgenic plants to produce food or medicines is not harmful to human health or the environment*.
*Dimension 3* (5% of the total inertia): the categories that most contribute to the inertial of this dimension are *negative or neutral attitudes toward biotechnology*, *biosafety, and genetic engineering* and *awareness regarding the use of transgenic plants to produce medicines*.

**Figure 1:**
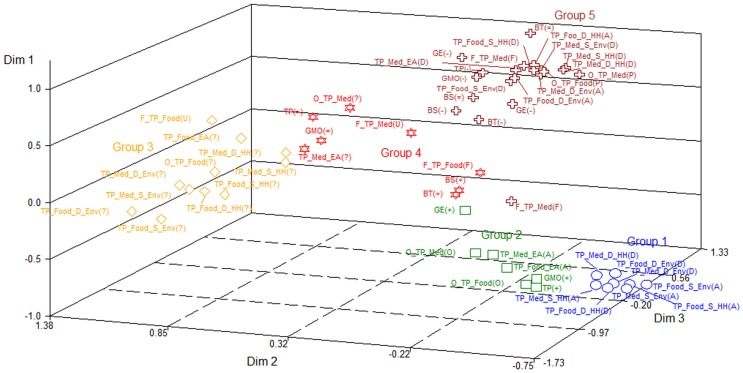
**Scatter plot of the categories of analysis in the three main dimensions of the multiple correspondence analysis**. Names for variables follow the list in Appendix 2 in Supplementary Material. (+) Positive; (−) Negative; (=) Neutral; (F) Familiar; (U) Unfamiliar; (O) Optimistic; (P) Pessimistic; (A): Agree; (D) Disagree; (?) Don’t Know/undecided.

In summary, Dimension 1 clearly differentiates positive or negative perceptions and expectations about agricultural biotechnology; Dimension 2 differentiates neutrality and the unawareness of GMOs; and Dimension 3 differentiates other views (use of GMOs to produce medicines) versus negative or neutral opinions regarding biotechnology. Figure 1 presents a more comprehensive picture of these patterns of association, with the geometric dispersion of the categories of analysis in the three main dimensions of the MCA. Categories in approximately the same direction from the origin and in approximately the same region of the Euclidean space are associated with each other.

The locations of both the categories and the individuals in the Euclidean space represented by Figure 1 were then used as criteria for the CA. Five groups were selected, which represented 78% of the total variability of the dimensions. Based on the patterns of association between categories of analysis and individuals, we interpreted the groups as follows:

*Group 1* – extremely positive attitude (219 individuals, 15% of the total): individuals associated with categories of positive perceptions of GMOs. Over 90% of positive responses toward the terms: biotechnology, biosafety, transgenic plants, GMOs, and genetic engineering. Almost all of the respondents describe themselves as aware of the use of transgenic plants for food and medicine production, and are optimistic about these practices, believing that the practices are not harmful for the environment and human health, and are ethically acceptable;
*Group 2* – positive attitude (307 individuals, 21% of the total): individuals associated with categories of positive and neutral perceptions of GMOs. Although the majority of respondents (over 70%) have positive attitudes toward the terms: biotechnology, biosafety, transgenic plants, GMOs, and genetic engineering, as well as an optimistic view of the use of transgenic plants to produce food and medicine, a considerable share of the respondents in this group describe themselves as unaware of the use of transgenic plants to produce medicines (40%) and are undecided about the risks that transgenic plants present to the environment and human health (between 25 and 45%);
*Group 3* – intermediate attitude (211 individuals, 15% of the total): individuals characterized by an indecisiveness, neutrality, and lack of knowledge regarding the use of GMOs. Although respondents tend to present a positive attitude toward the terms: biotechnology, biosafety, and genetic engineering (75% or more), this group is neutral with regard to the terms transgenic plants and GMOs (46%). Moreover, individuals in this group show the highest percentage of unawareness regarding the use of transgenic plants to produce medicine (62%) and food (18%). They tend to be undecided about the safety and ethics of using transgenic plants to produce food or medicines;
*Group 4* – negative attitude (159 individuals, 11% of the total): besides being associated with neutrality, indecision, and unawareness, these individuals are also intermediately associated with negative attitudes toward GMOs. Although 70% or more of the respondents have a positive attitude toward the terms: biotechnology, biosafety, and genetic engineering, 39% had a negative attitude toward the terms transgenic plants and GMOs. This group is also characterized by the unfamiliarity regarding the use of transgenic plants to produce medicines (62%);
*Group 5* – extremely negative attitude (543 individuals, 38% of the total): individuals associated with the most negative perceptions of GMOs. This group presents the highest percentage of negative responses for the terms: biotechnology, biosafety, transgenic plants, GMOs, and genetic engineering (between 19 and 85%). This group is also pessimistic about the use of transgenic plants to produce medicines (59%) and, above all, food (94%). They believe that these uses pose risks to human health and the environment.

### Patterns of Association

Table 2 presents the socioeconomic characteristics of the groups of attitudes toward GMOs. First, results highlight a higher prevalence in the group of men with the most positive attitude toward GM (Group 1 with 65% of men). Women, however, tend to be concentrated in the intermediate groups, especially groups 3 and 4. Thus, where women tend to have a more neutral attitude toward the use of GM crops, men tend to take more extreme positions, particularly with regard to showing positive attitudes. Gender differences in attitude toward biotechnology were also observed by Simon (2010).

**Table 2:** **Socioeconomic characteristics of the groups of attitudes**.

Characteristic	Groups					Total
	1	2	3	4	5	
*n*	219	307	211	159	543	1439
%	15	21	15	11	38	100
Region (% column)						
North	2	3	2	6	5	4
Northeast	5	6	7	13	13	10
Southeast	49	57	63	51	49	53
South	21	17	11	17	20	18
Midwest	22	17	17	13	12	15
	χ^2^ = 58.8*					
Education (% column)						
Superior	92	87	89	84	87	88
Middle	7	13	10	15	13	12
Fundamental	1	0	0	1	1	1
	χ^2^ = 8.8*					
Gender (% column)						
Female	35	47	57	53	43	46
Male	65	53	43	47	57	54
	χ^2^ = 27.0*					
Age (% column)						
<25 years old	14	27	17	28	22	22
25–34	32	35	37	30	35	34
35–44	20	15	22	18	18	18
>44 years old	34	22	23	23	26	25	
	χ^2^ = 27.7*					
Occupational status (% column)						
Managers	4	3	5	1	5	4
Professionals	76	65	60	64	64	65
Technicians	2	6	6	5	5	5
Service workers	4	5	10	4	8	7
Non-qualified workers	2	1	1	1	2	1
Students	12	17	15	24	13	15
Retired	0	2	3	2	3	2
	χ^2^ = 49.4*					

*Significant at the 95% confidence level, *p*-value <0.05.

There is no significant difference in the levels of education among the groups, but attitudes and perceptions toward GMOs seem to be significantly related to age. The most meaningful result is the relative concentration of people over 44 years of age in the group with the most positive attitudes toward GMOs. Young people (under the age of 25) tend to be concentrated in the group with negative attitudes toward GMOs (Group 4).

Moreover, the prevalence of positive attitudes is higher in the Midwest, the youngest prominent agricultural producer region in Brazil, and is lower in the Northeast, the oldest agricultural producer and characterized by the lowest level of productivity. Results also suggest that qualified professional workers tend to have extreme positive attitudes toward GMOs. On the other hand, students were more likely to be associated with a negative attitude (Group 4).

There were also significant relationships between the groups of attitudes and the trusted information sources that inform the public and monitor GMOs in Brazil (Table 3). For example, positive attitudes are directly related to confidence in the government, experts, and private companies as providers of information on transgenic plants and biosafety. In other words, the percentage of people who rely on these institutions tends to be higher in groups with more positive attitudes toward GMOs. By contrast, respondents with more negative attitudes toward GMOs tend to rely more on information provided by NGOs. Similarly, individuals with positive attitudes tend to hear about GMOs and biosafety from the government, experts, and private corporations, whereas those with negative attitudes tend to get their information from NGOs.

**Table 3:** **Composition of groups regarding trust and familiarity**.

Question	Groups					Total	χ^2^
	1	2	3	4	5		
Trusted information on GMO sources (%)							
Government	42	39	27	28	19	29	61.4*
NGO	14	28	35	38	57	39	146.8*
Experts (scientists)	95	95	89	76	55	77	263.3*
Media	14	17	13	15	13	14	3.4
Corporations	37	25	13	11	5	16	146.7*
Trusted information on biosafety sources (%)							
Government	45	36	25	23	22	25	115.1*
NGO	13	24	33	38	56	37	163.5*
Experts (scientists)	96	94	85	73	51	74	283.3*
Media	12	15	11	15	10	12	55.9*
Corporations	37	20	12	9	1	13	191.7*
Had heard discussions about transgenic plants and biosafety (%)							
Government	55	53	43	47	48	49	8.4
NGO	58	60	54	65	82	67	88.0*
Experts (scientists)	84	80	66	68	69	73	32.6*
Media	82	89	85	89	81	84	14.4*
Corporations	56	46	35	38	42	44	24.5*
Familiarity with the Brazilian biosafety authority							
Yes	88	73	52	60	75	72	83.6*
Knowledge of the name of this authority							
Yes	74	52	32	36	59	53	101.6*

*Significant at the 95% confidence level, *p*-value <0.05.

Results also highlight that positive attitudes toward GMOs are associated with the familiarity of the national committee that approve and disapprove GMOs in Brazil, as well as knowledge of its name. The highest percentage of people who claim to know about this Brazilian biosafety authority, as well as the people who actually know its name, is observed in the group with the most positive attitudes toward GMOs (Group 1). The lower percentages regarding the familiarity with this committee are witnessed in the intermediate groups, which have higher percentages of people who are undecided about their opinion of GMOs.

## Discussion

This study used a communication-based assessment tool in order to design a strategic communication plan that would promote public awareness and stimulate the debate on GMOs and biosafety. The fact that most respondents presented an agricultural bias would not affect the analysis used to guide a sound communication strategy, given that there will always be a connection between the means of communication and agricultural and food production.

The results obtained highlighted that respondents hear about GMOs and biosafety through the media (TV, radio magazine, etc.), although rely less on these means. Information is considered more reliable when supplied by scientists and experts. Although the majority of respondents have a university degree, the results obtained in this study are in agreement with data from other studies, such as Vogt and Polino (2003) where they found high levels of confidence in Brazilian science and scientists. An online survey conducted in 18 countries, including Brazil, also indicates that the credibility of science and scientists tends to be high.^5^

Respondents tend to view the terms biotechnology, biosafety, and genetic engineering more positively in comparison with transgenic plant and GMO. The disparity between respondents’ views in relation to technologies based on closely related scientific knowledge – biotechnology – is most likely due to the controversy that has arisen from transgenic plants being used for food, which is consistent with the literature review (Soares, 2003). Eating is a necessary risk, which means it is involuntary, broad, and unknown. However, using biotechnology for drug production has potential benefits that are tacitly understood by the respondents, especially those who are more aware of science and technology.

Multivariate analysis highlights many ways to improve public awareness. GMOs for food production is in the spotlight, meaning that it is the main source of disagreement among respondents, as opposed to biotechnology in general. The groups with positive attitudes toward biotechnology, primarily that of transgenic crops, are predominantly composed of males, senior citizens, and individuals who trust in experts as sources of information, even regarding biosafety. By contrast, the groups of respondents with negative attitudes toward GM crops tend to be young people who are under the age of 25, particularly students. These groups primarily consist of individuals who rely on NGOs as an information source about GMOs and biotechnology.

Our findings show a polarization between groups of respondents with positive attitudes and a certain degree of awareness of GMOs, and those with neutral or negative attitudes and lower awareness of GMOs. Since many of the members of these latter groups are unfamiliar with the use of GMOs, communication on the subject is targeted at these members in order to improve the level of knowledge on GMOs in Brazil. Moreover, the importance that people place on scientists suggests that there is a need for a sound strategy of communication that combines media resources with scientifically qualified and accessible knowledge to ensure trust and confidence.

Public institutions involved in GMOs research in Brazil are numerous and important. According to our results, these institutions have an important function as source of scientific information, and policy makers should ensure the dissemination of this knowledge. The most appropriate communication strategy should be developed based on the public’s perception and its needs.

Although our findings also tend to explain the limited utility of the “scientific literature,” it is recommended that the Brazilian Government focus more on the development and dissemination of scientific research oriented to the public’s perception of a particular technology, prior to the deployment of this technology.

The debate on adopting GMOs demands the establishment of good channels of communication amongst different types of stakeholders with appropriated means to discuss issues related to science and public views. Confidence and transparency are necessary for the sustainable strengthening of public opinions and perceptions regarding the introduction of a technological innovation. The potential discrepancies between attitudes (which were measured in this study) could contribute to further investigations based on the actual behavior of consumers that would be useful to strengthen the communication approach employed.

## Authors Contribution

All authors contributed equally to this work.

## Conflict of Interest Statement

The authors declare that the research was conducted in the absence of any commercial or financial relationships that could be construed as potential conflicts of interest.

## Footnotes

^1^http://www.lacbiosafety.org

^2^www.embrapa.br

^3^As per the Brazilian National Commission on Ethics in Research – CONEP/CNS/MS, the commission analysis/permit for research of this kind is not required. CONEP website: http://conselho.saude.gov.br/Web_comissoes/conep/index.html

^4^Available at www.ibge.gov.br. Accessed in September, 2013.

^5^Published in September 2010. Taken from http://www.revistapesquisa.fapesp.br/index.php?art=6744&bd=2&pg=1&1g
