# Supplementary material for: A Study of Stakeholder Views to Shape a Communication Strategy for GMO in Brazil
Source: Front Bioeng Biotechnol. 2015 Nov 9;3:179. doi: 10.3389/fbioe.2015.00179 (PMC4638152; doi:10.3389/fbioe.2015.00179)
Supplement: Supplementary file 1 [file Data_Sheet_1.DOC]

# **Supplementary material for**

**Experiences on communicating biosafety in Brazil: lessons toward a good communication strategy**

Deise Maria Fontana Capalbo^*1^, Olivia Márcia Nagy Arantes^1,2^, Alexandre Gori Maia^3^ , Izaias Carvalho Borges^3^, Jose Maria Ferreira Jardim da Silveira^3^

^1^ Embrapa Environment, Brazilian Corporation of Agricultural Research, Jaguariúna, São Paulo State, Brazil

^2^ Independent Consultant, Jaguariúna, São Paulo State, Brazil

^3^ Institute of Economics, State University of Campinas, Campinas, São Paulo State, Brazil

**Correspondence**

Dr Deise M. F. Capalbo

Embrapa Environment

Rodovia SP 340 - km 127.5

Jaguariuna / SP

CEP 13820-000

Brazil.

[deise.capalbo@embrapa.br](mailto:deise.capalbo@embrapa.br)

##

**This supplement includes**  2 Appendix

**APPENDIX 1**

**Translation of the online survey applied for the present study purposes.**

**Public perception of transgenic plants in Brazil**

LAC Biosafety is an international project on biosafety of transgenic plants being developed with funds from the Global Environment Facility - GEF, to strengthen technical capacity and public perception, and to generate information that will serve as a reference for biosafety actions, according to the Cartagena Protocol. The project is being developed in a consortium formed by four countries, including Brazil. The Brazilian coordination is under the responsibility of Embrapa Environment. It is known that there is a controversial dialogue and controversial information regarding the transgenic plants and their safe use. Therefore, one of the project goals is to know the public perception of transgenic plants and their regulations. Answer the questionnaire below so that your questions can be answered in the site www.lacbiosafety.org by the experts participating in the project. If you prefer to receive the questionnaire by mail, please call Embrapa Environment *(specific telephone number indicated in the original questionnaire).*

Thank you for participating.

**QUESTIONNAIRE** (*) = MANDATORY

*** Education** ***Choose just one of the following:***

3rd Degree

2nd Degree

1st Degree

**Profession** (open question)

**Age** (open question)

*** Sex**

Female

Male

*** City/State** (open question)

**If you want to know the result of this research, register an email address** (open question)

**1. Fill with the correspondent numbers the words below:**

**(1) if you have positive image**

**(2) if you have negative image and**

**(3) if you have neutral image:**

Biotechnology ( )

Biosafety ( )

Transgenic plants ( )

Genetically Modified Organism ( )

Genetic Engineering ( )

*** 2. The use of transgenic plants for the production of medication you:** *Choose just one of the following*

Know

Do not know

*** 3. When it is about the use of Transgenic Plants for the production of medicines you are:** *Choose just one of the following*

Optimistic

Pessimistic

Undecided

*** 4. The use of transgenic plants for the production of food you:** *Choose just one of the following*

Know

Do not know

*** 5. When it is about the use of Transgenic Plants for food you are:** *Choose only one of the following*

Pessimistic

Optimistic

Undecided

**6. Mark the item in the column expressing your opinion. Use of Transgenic Plants in the production of drugs is:**

*Options I agree I disagree I do not know*

Not harmful to the environment

Not harmful to human health

Dangerous to the environment

Hazardous to health

Ethically acceptable

*** 7. Check the item in the column expressing your opinion. Use of Transgenic Plants in food production is**

*Options I agree I disagree I do not know*

Dangerous to the environment

Hazardous to health

Not harmful to the environment

Not harmful to human health

Ethically acceptable

*** 8. You know the Cartagena Protocol which Brazil is a signatory?** *Choose just one of the following*

Yes

No

Partly

**9. Check your level of trust in organizations or in persons named below as a source of information about what Transgenic Plant is:**

*Options: Trust Do not trust Indifferent*

Government

Non-Governmental Organizations - NGO

Scientist Specialist

TV, Radio, Newspaper, Magazine, Internet

Private Companies

School/teachers

Doctor

Governmental International Organizations

Church

**Comment your choices in question 9** (open question)

*** 10. Check your level of trust in organizations or in persons named below as a source of information on Transgenic Plant Biosafety (risks and benefits).**

*Options: Trust Do not trust Indifferent*

Government

Non-Governmental Organizations - NGO

Scientist Specialist

TV, Radio, Newspaper, Magazine, Internet

Private Companies

School/teachers

Doctor

Governmental International Organizations

Church

**Comment your choices in question 10** (open question)

*** 11. Check which group(s) or person(s) below you see/hear informing the public about Transgenic Plants.** *Choose one or more alternatives*

Government

NGO

Scientist Specialist

Scientist

TV

Radio

Newspaper Magazine

Private Companies

School

Doctor

Governmental International Organizations

**Other (option related to the issue 11) -** (open question)

**12. Check the column expressing your opinion. The published scientific information on Transgenic Plants is:**

*Options I agree I disagree I do not know*

Clear

Conflicting

Reliable

Inadequate

**Comment your choices in question 12** (open question)

*** 13. You know that there is a Commission to approve/disapprove the release of Transgenic Organisms in Brazil?**

Yes

No

**For question 13, if Yes, describe which:** (open question)

*** 14. Do you know what are the organizations/institutions that participate of the Commission cited in the previous question?** *Choose just one of the following*

Yes

No

In part

*** 15. Check the information you want to access or the subject, in addition to those already available in the project site.** *Choose one or more alternatives*

Benefits

Existing and responsible regulation

Ethics of the debate

Processes and techniques used

Who finances

Risks

I am not interested in the subject

Biodiversity

**Other (option related to the question 15)** (open question)

**Appendix 2**

Variables used in the questionnaire

| **Name** | **Description** | **Response** |
| --- | --- | --- |
| *Section 1 - Socioeconomic variables* | |  |
| Educ | Education | Superior, Middle, Fundamental |
| Occup | Occupation | Open-ended |
| Age | Age | Open-ended |
| Gender | Gender | Male, Female |
| City | City | Open-ended |
| *Section 2 - Perception and Awareness of GMO and Biosafety* | |  |
| BT | Perception of biotechnology | Positive, Negative, or Neutral |
| BS | Perception of biosafety | Positive, Negative, or Neutral |
| TP | Perception of transgenic plants | Positive, Negative, or Neutral |
| GMO | Perception of genetically modified organisms | Positive, Negative, or Neutral |
| GE | Perception of genetic engineering | Positive, Negative, or Neutral |
| TP_Med | The use of transgenic plants to produce medicine | Familiar or Unfamiliar |
| TP_Med | The use of transgenic plants to produce medicine | Optimistic, Pessimistic, or Undecided |
| TP_Med_S_Env | The use of transgenic plants to produce medicine is not harmful to the environment: | Agree, Disagree, or Don’t know |
| TP_Med_S_HH | The use of transgenic plants to produce medicine is not harmful to human health: | Agree, Disagree, or Don’t know |
| TP_Med_D_Env | The use of transgenic plants to produce medicine is dangerous to the environment: | Agree, Disagree, or Don’t know |
| TP_Med_D_HH | The use of transgenic plants to produce medicine is dangerous to human health: | Agree, Disagree, or Don’t know |
| TP_Med_EA | The use of transgenic plants to produce medicine is ethically acceptable: | Agree, Disagree, or Don’t know |
| TP_Food | The use of transgenic plants to produce food | Familiar or Unfamiliar |
| TP_Food | The use of transgenic plants to produce food | Optimistic, Pessimistic, or Undecided |
| TP_Food_S_Env | The use of transgenic plants to produce food is not harmful to the environment | Agree, Disagree, or Don’t know |
| TP_Food_S_HH | Using transgenic plants to produce food is not harmful to human health | Agree, Disagree, or Don’t know |
| TP_Food_D_Env | The use of transgenic plants to produce food is harmful to the environment | Agree, Disagree, or Don’t know |
| TP_Food_D_HH | The use of transgenic plants to produce food is harmful to human health | Agree, Disagree, or Don’t know |
| TP_Food_EA | The use of transgenic plants to produce food is ethically acceptable | Agree, Disagree, or Don’t know |
| *Section 3 – Trusted Information Sources and Familiarity with the Brazilian Biosafety Authority* | |  |
| Gov_GMO | Trust in the Government as source of information on GMO | Trust, Don't trust, Indifferent |
| NGO_GMO | Trust in the NGO as source of information on GMO | Trust, Don't trust, Indifferent |
| Exp_GMO | Trust in the Experts(scientists) as source of information on GMO | Trust, Don't trust, Indifferent |
| Media_GMO | Trust in the Media as source of information on GMO | Trust, Don't trust, Indifferent |
| PC_GMO | Trust in the Private Corporations as source of information on GMO | Trust, Don't trust, Indifferent |
| Gov_BS | Trust in the Government as source of information on biosafety | Trust, Don't trust, Indifferent |
| NGO_BS | Trust in the NGO as source of information on biosafety | Trust, Don't trust, Indifferent |
| Exp_BS | Trust in the Experts(scientists) as source of information on biosafety | Trust, Don't trust, Indifferent |
| Media_BS | Trust in the Media as source of information on biosafety | Trust, Don't trust, Indifferent |
| PC_BS | Trust in the Private Corporations as source of information on biosafety | Trust, Don't trust, Indifferent |
| GOV_TP | Ever heard the Government talk about transgenic plants | Yes, No |
| NGO_TP | Ever heard the NGO talk about transgenic plants | Yes, No |
| Exp_TP | Ever heard the Experts (scientists) talk about transgenic plants | Yes, No |
| Media_TP | Ever heard the Media talk about transgenic plants | Yes, No |
| PC_TP | Ever heard the Private Corporations talk about transgenic plants | Yes, No |
| K_Comission | Familiarity with the Brazilian biosafety authority | Yes, No |
| K_Name | Knowledge of the name of this authority | Yes, No |
